# The Impact of Invisibility on the Health of Migrant Farmworkers in the Southeastern United States: A Case Study from Georgia

**DOI:** 10.1155/2012/760418

**Published:** 2012-07-01

**Authors:** Kari M. Bail, Jennifer Foster, Safiya George Dalmida, Ursula Kelly, Maeve Howett, Erin P. Ferranti, Judith Wold

**Affiliations:** Nell Hodgson Woodruff School of Nursing, Emory University, 1520 Clifton Road, Atlanta, GA 30322, USA

## Abstract

Migrant farmworkers represent one of the most marginalized and underserved populations in the United States. Acculturation theory cannot be easily mapped onto the transnational experience of migrant farmworkers, who navigate multiple physical and cultural spaces yearly, and who are not recognized by the state they constitute, “the Citizen's Other” (Kerber, 2009). This paper utilizes narrative analysis of a case study to illustrate, through the relationship of the narrator to migrant farmworkers and years of participant observation by the coauthors, how isolation from family and community, as well as invisibility within institutions, affect the health and well-being of migrant farmworkers in southeastern Georgia. Invisibility of farmworkers within institutions, such as health care, the educational system, social services, domestic violence shelters, and churches contribute to illness among farmworkers. The dominant American discourse surrounding immigration policy addresses the strain immigrants put on the social systems, educational system, and the health care system. Nurses who work with farmworkers are well positioned to bring the subjective experience of farmworkers to light, especially for those engaged with socially just policies. Those who contribute to the abundant agricultural produce that feeds Americans deserve the recognition upon which social integration depends.

## 1. Introduction

The agricultural industry in the Southeast of the United States is an important contributor to the economy of the region. The industry relies on the manual labor of farmworkers who plant and harvest crops, work in packing houses, processing plants, and preparation facilities associated with farms [1]. The majority of farmworkers in the southeastern United States are migrants from Latin America, with some representation from other regions [2]. A migrant farmworker is defined as an individual whose principal employment (at least 51%) is in agriculture on a seasonal basis and lives in temporary housing (US Code, Public Health Services Act, “Migrant Health”). Currently, there are over 3 million migrant farmworkers in the United States [1]. In 1964, the USA federal H-2A program was created to meet agricultural production needs. The H-2A temporary agricultural program enables farmers who are unable to recruit sufficient domestic workers to bring foreign workers to USA to perform agricultural labor or services of a temporary or seasonal nature. Legal protections that apply to these H-2A workers are enforced through the Wage and Hour Division of the United States Department of Labor [3].

The state of Georgia in the United States has a robust agricultural industry. For example, in one county alone, Riverside County, there are over 600 farms (Riverside County is a pseudonym, for confidentiality reasons.). Many farms rely on human labor from farmworkers to plant and harvest the crops. Riverside County, an agriculture intensive region in southern Georgia, has over 600 farms, of which just 3 are H-2A farms. The balance of farms in Riverside County is staffed by undocumented farmworkers. Up until 10 years ago, there was just 1 H-2A grower in the state of Georgia. The rights of farmworkers and responsibilities of farmers are delineated in the Migrant and Seasonal Agricultural Worker Protection Act (MSPA), which apply to both H-2A workers as well as undocumented workers. The MSPA requires housing inspection and compliance with federal and state safety and health standards, provides itemized statements of earnings and deductions, and ensures that vehicles for worker transportation meet federal and state safety standards and insurance requirements, and that each driver is properly licensed. The goal of these regulations is to protect all farmworkers from substandard and dangerous conditions.

Farmworkers in Georgia usually arrive via Mexico, and they include not only Mexicans (the majority), but Central and South Americans, as well as some persons from the Caribbean. Farmworkers in southern Georgia are typically undocumented, and farmers rely on smugglers, known as coyotes, to shuttle them back and forth from Mexico to Georgia. Farmworkers come to Georgia looking for economic opportunities, but frequently undocumented workers' ability to be financially independent is untenable given the debts owed by farmworkers to a contractor. The contractor typically finances the farmworker's travels and living expenses, while expecting full reimbursement plus interest before the farmworker leaves his/her job [1].

In 1996 the Health Centers Consolidation Act established a centralized funding source for Migrant/Seasonal Farmworker Health Centers, as well as health centers for other vulnerable populations. Today there are 154 migrant farmworker health centers in 42 of the United States, which provide health care to over 807,000 farmworkers [4]. Despite these health centers, access to health care remains a serious challenge to farmworkers.

The major threats to migrant farmworker health documented in the literature include occupational injuries, pesticide exposure, infectious disease, heat stroke, and dermatological conditions [5]. The working and living conditions of migrant farmworkers generate unique health hazards, due to both occupational challenges as well as injury and illness that stem from the conditions imposed by the culture of migrant farmwork, including dependency and poverty [6]. For example, food insecurity is prevalent among farmworkers in the state of Georgia, with as many as 63% reporting inadequate food. Undocumented workers have an adjusted risk of food insecurity 3 times higher than H-2A workers [3]. While significant research has been conducted on occupational hazards of farmwork, such as pesticide exposure, injuries, and dermal conditions, there has been little focus on the differential health risks encountered by farmworkers resulting from their marginalization. Farmworkers differ from the general immigrant population in their relatively unhealthy and unacculturated status. They also experience elevated rates of social and economic discrimination compared to the general immigrant population of the USA [5].

Multiple studies have been done of anxiety and depression among farmworkers [7–13]. Joseph Hovey has written several articles on migrant farmworker health that highlight associations between high levels of depression and various risk factors, including family dysfunction, ineffective social support, hopelessness, and high acculturative stress. While there appears to be agreement among scholars that acculturation leads to stress and increased incidence of mental health dysfunction, one study found that the prevalence of psychiatric disorders among migrant farmworkers was lower than for Mexican Americans and the US population as a whole, but increased with further acculturation and primary residence in the United States [12].

Although debates about migrant workers have emerged across the country, they are particularly volatile in the state of Georgia. As of July 1st, 2011, Georgia House Bill 87, The Illegal Immigration Reform and Enforcement Act of 2011, was enacted with the goal of deterring the undocumented from entering the state (House Bill 87). The law requires that businesses and government agencies check the immigration status of new workers through a national system and impose fines of up to $250,000 and 15 years in prison on those who use false identification. An additional provision requires people applying for food assistance and public housing to provide specific forms of identification [14]. The state's sixty-nine billion dollar a year agricultural industry is at stake with the passage of this new immigration law [15]. The purpose of this paper is to inform public debate by presenting empirical data about the scope of health challenges facing migrant farmworkers. Nurses play an important role in the care of migrant farmworkers in USA, but many more nurses are needed to care for farmworkers. This paper utilizes narrative analysis of a case study to illustrate, through the relationship of the narrator to migrant farmworkers and the participant observations of the co-authors, how isolation from family and community, as well as invisibility within institutions, impact the health and well-being of migrant farmworkers.

### 1.1. Theoretical Framework

Studies of migration have often been framed by “acculturation theory” also known as “assimilation theory.” Acculturation theory is based on the assumption that immigrants succeed by adopting cultural practices of their host country and relinquishing those from their homes [22]. Reference [4] Theories of migration and health have held explanatory power for permanent—and not temporary—migration [1]. A 1997 review of acculturation theory, tracing its evolution and widespread critique, acknowledges the theory's absence of *recognition* for the diversity of modern day immigrant populations from the global South. Alba and Nee discuss the weakness of acculturation theory in that “a more differentiated and syncretic conception of culture is needed, and a recognition that American culture was and is more mixed, much more an amalgam of diverse influences, and that it continues to evolve.” [16, page 834]. Nevertheless they define assimilation as the decline and ultimate disappearance of an ethnic/racial distinction with the social and cultural differences that express it. The idea that assimilation involves the disappearance of ethnic and racial distinction assumes recognition of individual subjectivity.

 Critical migration discourse obviates acculturation theory by the interrogation of the construct of citizenship. Citizens are recognized members of the state, entitled to protection, rights, material support, and a degree of political loyalty. Noncitizens, which include undocumented, or illegal persons are not so recognized [17]. The binary category of citizen/noncitizen serves to perpetuate the invisibility of non-citizens. States are called to contain and steward increasingly scarce resources. Thus, the effect of perpetuating migrant farmworkers in a stateless category, as “the Citizen's Other” [18, page 76] addresses its economic need in the contemporary global structure. Nevertheless, the political desire to create boundaries between foreigners and full citizens, especially with respect to limited resources, such that health care is uneasily juxtaposed to the economic need for the productive migrant labor that non-citizens provide [19]. In addition to a legal obligation to ensure human rights within their borders, states have an economic interest in healthy workers who are physically and mentally able to work.

Recognition (or its absence) is a key concept in contemporary critical social theory. Recognition has its origin in the phenomenology of consciousness, in Hegelian philosophy, which constitutes social reality as a relationship between subjects, in which each sees the other as an equal, but also separate. In order to become an individual subject, one must recognize, and be recognized by, another subject [20, page 10]. “All social integration depends on reliable forms of mutual recognition…which can be regarded as the engine of social change” [20, page 245].

Since 1993, a partnership between health professional students and faculty members from several academic institutions in the state of Georgia and a farmworker health clinic in south Georgia has provided health care to migrant and seasonal farmworkers. This project, The Farmworker Family Health Program (FWFHP), brings students and faculty to the schools migrant children attend, as well as to the fields and barracks where farmworkers live and work to provide basic health care and screening services [21]. This partnership has made it possible to develop a deeper understanding of the experience of migrant farmworkers in this setting, which led to the current study.

## 2. Materials and Methods

Studying transient populations presents numerous methodological obstacles. Frequent relocation challenges researchers to gain access to this population. In addition, there is understandable fear among undocumented farmworkers that exposure may result in fines, incarceration, and deportation. Farmers may have incentive to keep farmworker barracks hidden and inaccessible, as their business could be penalized for employing undocumented workers.

In 2010, the director of the farmworker health facility in the FWFHP and one of the co-authors attended a workshop together to develop a proposal for community-based participatory research with farmworkers, sponsored by the United States National Institutes of Health (NIH). The farmworker health facility director, a middle aged Caucasian woman whom we refer to with the pseudonym Jackie, presented 5 critical health issues facing the migrant farmworker population (lack of prenatal care, alcoholism, domestic violence, poor dental care, and diabetes). The NIH faculty quickly identified Jackie to be a key informant, a source of enormous knowledge about the lives of farmworkers, because she has lived amongst and worked with farmworkers in the same site for more than twenty years. The same faculty encouraged the FWFHP partnership to tap into Jackie's knowledge about the experience of migrant farmworkers in her setting. Such knowledge might usefully inform policy makers and others about this little understood population.

As a result of the workshop, the authors (six of whom are faculty in the FWFHP) conducted in-depth interviews with Jackie over three months in 2010, to elicit a deeper description of the lives of farmworkers related to the critical health issues she had identified. Additionally, all the authors have been participant observers (ranging from a few days to eighteen years) at the site, as nurses who provide health clinics, health education, and referral.

Through years of advocacy and membership in the Mexican community, Jackie has earned the trust of the migrant farmworker community of southern Georgia. Jackie was married to a Mexican and has raised children in the Mexican community. She has personal and professional relationships with farmworkers and is clearly devoted to their well-being. Jackie was on the advisory council for the migrant health clinic for 8 years before she was offered the clinic director position. She is well-known and trusted within the migrant community in which she lives by both farmworkers, and farmers alike, and is an invaluable resource for migrant health.

The data analyzed here consist of 5 in-depth, in-person interviews conducted with Jackie over several weeks in the summer of 2010. Interviews were conducted either at Jackie's house or in her car, while driving around the area, visiting farm fields and packing areas, as well as farmworker housing. The project was submitted to the Institutional Review Board (IRB) of the main university partner; they ruled that the project didn't require IRB review. The resulting interviews were transcribed by a professional transcription service. Analysis of the transcripts of these interviews was done utilizing open coding and making memos, techniques possible with MAXQDA software. Memos were used to generate ideas and engage with the narratives. From these memos, codes were created, and ultimately several of the codes that were interrelated and most engaged with the themes developed in memos were selected for analysis.

## 3. Findings and Discussion

### 3.1. Invisibility and Isolation

 While farmworkers provide the manual labor required to produce the fruits and vegetables Americans see every day in the supermarket, they are largely both figuratively and literally invisible. Driving along a rural route in Southern Georgia, a passerby cannot see the hundreds of men and women, stooped among the pepper plants, from the road. In the following passage from an interview conducted while driving through the fields, Jackie explains

*“…there is one church that we [Farmworker Family Migrant Health Project volunteers] go to for lunch and when we first started going there, the minister at that church… told the group of nurses that the first time we went to lunch there that he didn't know there were migrant farm workers here, and see, they are oblivious to them. They ride right by them. I guarantee if we stopped at any house up there and said, “I am looking for a farm labor camp close by here? Do you know where it is?” They wouldn't know this was here. They wouldn't know this was here.”*

Throughout these interviews, Jackie tells the story of fragmentation of the farmworker family. Migrant farmworker children are impacted by invisibility, from their entry into the United States to their interactions with state institutions. Children of undocumented workers may cross the border with their parents and traverse the desert or rivers, while Mexicans with more social capital pay others to pass them off as their own children and travel with them. Crossing children is more dangerous when the children can talk, but do not understand why they are not allowed to talk. Once children arrive with their parents, they often fall through the cracks of the public school system if they are enrolled in school. While the parents do not need to show proof of legal residence to enroll their children in school, they must have a birth certificate, which the parents may not have brought, or may have lost, during the trip to the United States.

 According to Jackie, if migrant farmworker parents are able to locate a daycare, there is a good chance that their child would not be able to enroll. Daycares would rather have children enrolled whose living situation is stable, so the daycare can have a guaranteed revenue stream. Additionally, there is often minimal bilingual staff.

*“You know, I don't think it's that they [daycares] don't want [migrant] children necessarily, but they don't have the… if it's a licensed daycare, they don't have the provisions in place and so you know they would be in violation [of rules about accomodations for non-English speakers] and they don't know how to do that sort of thing. They don't have anybody to translate their paperwork or anything like that. They don't have anybody that can converse with their family. That sort of thing. So you know it's, it's a combination of things…(Sic)”*

Jackie's narratives also chronicle the fundamental instability of farmworker employment, as crop yields are highly dependent on unpredictable environmental factors; hence, there may be a flood, a tornado, or a freeze that affects the crop yield and forces farmworkers to relocate. Farmworkers are at the mercy of the harvest for their livelihood, as they do not receive unemployment benefits. According to Jackie, in the event of crop failure, H-2A farmworkers do not receive relocation money or disaster assistance from the federal government, while there is crop insurance for farmers. Additionally, H-2A farmworkers do not typically receive unemployment, so they must follow the crops to sustain their livelihood. Jackie describes below how timing is essential in the agricultural industry.

*“During peak season [is] when they're picking squash, tomatoes, cucumbers, eggplant, those kinds of things. You can walk out in the field and see them. Let's say, the cucumbers are here. And they thought they're not quite ready to be picked, but tonight if the humidity is ideal, we better wake up in the morning and they be ready, they grow over night. Cucumber, zucchini, squash, can grow overnight, can be ready in the morning when you wake up. That's why you need a labor force when you are a big grower like this. Every one of those plants is hand planted. The plastic is laid by machine, but there are people following pushing dirt up on it to keep it from blowing up. Every one of those stakes, all that stringing is done. And talking of occupational health, when the stringing is going on, we have a lot of rotor cuff injuries because they're doing this all day long up and down these rows.”*

Invisibility takes a toll on the health of farmworkers. Jackie describes that when they are sick, farmworkers do not have the traditional support network of family to care for them or make sure that they take care of chronic health conditions. Not only is farmworker livelihood intimately connected to the weather and the seasons, Jackie explains that it is also inextricably connected to their health. The types of crops harvested determine the variety of pesticides used, which in turn may differentially impact their health. Certain repetitive movements elicit specific types of overuse injuries, such as rotator cuff injuries. While picking cucumbers, contact dermatitis is common among farmworkers. Cutting mustard greens with a butcher knife frequently results in laceration injuries.

Jackie was notified when a farmworker, very ill with syphilis, meningitis, and AIDS, was hospitalized and did not have anyone to care for him, or serve as his health care proxy as he was intubated and therefore unable to communicate. When he recovered, a girl claiming to be his sister-in-law picked him up from the hospital. It turned out that he had given a false name at the hospital, and he did not have a sister-in-law. This type of confusion and misrepresentation speaks to both the underlying fear of disclosing one's true identity, as well as a lack of real social support. Many farmworkers use false names and identification—they do not exist legally.

### 3.2. Access to Health Care

 Jackie's narratives underscore the lack of access to health care among migrant farmworkers. As she recounted, a migrant farmworker was living in his car, and he appeared constantly intoxicated. People who lived in the surrounding area complained of his intoxicated behavior, and so Jackie went to investigate. She found that the man was not drinking at all. She found money to send him to a doctor, and it was discovered that he had a brain tumor that was exerting pressure on his brain, so as to make him appear intoxicated. The tumor was operable, and once it was removed, the man's mental status was completely restored. Late diagnosis of this brain tumor resulted from lack of access to care. Delayed diagnosis of treatable conditions leads to an increased burden on the health care system, as well as unnecessary suffering by individuals and families. This is also the case with delayed diagnosis of pregnancy.

 Jackie explained the complicated situation surrounding pregnant migrant women. There are no laws regarding pregnancy and the H-2A visa program—women are not screened for pregnancy upon entering the country, and there are no consequences if a woman becomes pregnant during her contract. In fact, farmworker women are protected, as are American women, from being laid off because of their pregnancy. This results in women working under very challenging conditions during pregnancy, and yet as long as they continue to do so, their hours cannot be limited by employers. In addition, under the conditions of the H-2A contract, the woman must work and be paid for at least 75% of the time, and so taking the time off to obtain prenatal care may be especially difficult.

Jackie explained how state-funded programs in Georgia impact pregnant farmworker women. In the state of Georgia, pregnant farmworker women, whether documented or authorized to work, do not qualify for publicly funded healthcare in the USA (Medicaid). Emergency Medicaid is available to cover the delivery of pregnant farmworker women; however, their only prenatal care option is to enroll in a state-funded program, “Babies Born Healthy.” This program is commonly underfunded by the state legislature, leaving women without access to prenatal care during the last few months of the fiscal year when funds have been expended. While a farmworker's baby will be an American citizen when born in the United States, and thus entitled to state-sponsored programs, his/her mother is not entitled to any benefits under federal law. Additionally, there is a limited access to abortion among farmworker women. They would be required to pay out of pocket and also travel a long way to obtain an abortion.

 Jackie described a case where a 21-year-old woman had received no prenatal care. The father of the baby left as soon as she became pregnant. For the first 4 weeks after the child was delivered, she did not understand what was wrong with her baby and brought the newborn to the clinic. “She came in and sat down in my office…and she said, why is my baby's head so big?…Does my baby have a brain?” Jackie took the young mother and child to the pediatrician, who referred her to a specialist at Children's Medical in Atlanta. Finding a specialist who would accept this child as a patient was challenging, as the child's Medicaid coverage was still pending. The child was seen and was determined to have no brainstem. While Jackie discovered that this diagnosis had been made at birth, the young woman did not understand the meaning of the diagnosis or the choices she would have to make as a result. The young woman had received no prenatal care during her pregnancy. This young mother had come to Georgia on an H-2A visa, but overstayed it, making her current status undocumented. She had to move out of the barracks once she gave birth, and was living in a trailer with several other people, sleeping on a mattress on the floor.

In order to qualify, women must have photo identification. According to Jackie, many undocumented women in Southern Georgia obtain false identification using a name that is not their own. This presents a problem at delivery, when the wrong name might go on the birth certificate of the child. If the woman wants to return to Mexico with her child, unfortunately the only recourse is to obtain a DNA test in order to change the child's name, which is very expensive. Just as the woman might have had to be smuggled across the border to work in the United States, she may have to smuggle her own child back across the border. Jackie described the way that this can occur—the mother might pay another woman to use her baby's birth certificate.

In addition to the challenges of providing prenatal care to farmworker women, there is also the challenge of getting women to utilize prenatal care. They still have to pay for a portion of it that is significant in their overall income; at the Riverbend Clinic, it is $300 for prenatal care, charged at the first visit. Women have often seen their mothers and aunts go without prenatal care, and many wonder if it is a worthwhile expense.

 Access to health care includes not only physical access, but also whether health care is culturally and linguistically accessible to farmworkers. Another of Jackie's stories illustrates the cultural divide between migrant farmworkers and the American social welfare program. A Department of Family and Children's Services (DFCS) worker made a home visit to a young Guatemalan woman's home. The DFCS worker was getting very frustrated with the young mother and asked the woman what the baby had eaten that day. Jackie saw the pot of *caldo*, a traditional broth made from the meat of bones and fed to children, on the stove, and already knew the answer. She opened her cabinet and found that it was full to the brim with baby cereal, which she had never opened because she never understood what to do with it. The DFCS worker also insisted that the young woman obtains a crib for the safety of her child.

*“Well, when we came the next time, she very excitedly met us at the door and told me to tell her, “I have a crib.” Well, I was excited too. I said, “Oh, she has a crib” and so she's telling us to come, come with her, come with her, so we're following her to the back of the trailer and we get back there and what again, it's about paradigms, what you and I would see as a closet, this young girl saw as her room and she was very excited because she had been able to recall how her mother had created cunas [cradle] by creating this sling-type thing that the baby slept in. Almost like a hammock… And so to her, she had done exactly as she was taught. She had got a cuna, and the DFCS worker, before I could do anything, began ripping it out and just screaming at the top of her lungs about the baby being in a closet and the girl had absolutely no connotation of closet. Her home didn't have closets in Guatemala, you know, just like 100 years ago our houses didn't have closets and so she was devastated. She was crying. The baby was crying. The DFCS worker was yelling.”*

While the young mother was proud of herself for fulfilling the requirements of the DFAS worker and providing for her child, her concept of the *cuna*, or cradle, did not align with the American crib envisioned by the DFCS worker. Rather than supporting this mother's attempts to be a loving and attentive parent, the DFCS worker, representing the role of the state, shamed this young woman and made her feel like she was a poor parent and provider for her child.

Jackie recounted a story about a 15-year-old boy who tried to adjust to farmworker life and the painful isolation that often comes with solo migration. This boy was brought across the Mexican border in order to work on a farm in Florida, and a contractor brought him to South Georgia from Florida. He was all alone without family or friends. Several months after arriving in Georgia, the boy began to have panic attacks. One night, he had a panic attack so severe, he thought it was a heart attack, and he called 911 (emergency number) at the barracks where he lived, and an ambulance transported him to the hospital. Jackie was called by the Department of Family and Children's Services (DFCS) when the hospital realized that he did not have any relatives or guardians. She was asked to take him in, as DFCS had no foster families who would take him and nowhere else to bring him. She decided to take him, and found out that he was not free to go home to Mexico until he paid all of the money back to the contractor who had paid the coyote that crossed him (smuggled him across the border). The boy was concerned that he or his family would be hurt if he did not pay back the money he owed before returning to Mexico. Jackie used her connections to demand that one of the farm contractors pay the bus fare to return the boy to his home and promise not to hurt him or his family.

 This young man traveled alone, without family or friends, to Georgia, leaving behind his support network. Once farmworkers arrive in Southern Georgia, rather than starting with a clean slate and earning money to send back to their families in Mexico or support their families in the United States, many start with the burdens described in this case. While the living quarters are close, Jackie notes a lack of human connection among the male farmworkers, whose primary socialization revolves around alcohol once the workday is over. Alcoholism is a part of daily life of farmworkers, and alcohol is available even when food is not readily available, as alcohol vendors come around the barracks in trucks. As most farmworkers do not have access to their own transportation, they are unable to reliably go out to purchase food or do laundry; however, alcohol is readily available to farmworkers and children of any age.

Documentation status has a significant impact on the mental health of farmworkers. Access to mental healthcare is a challenge for all Americans, but it is particularly challenging for those who lack insurance, a language in common with most practitioners, and transportation and availability to access services. Unique mental health challenges arise from the circumstances of migrant farmworkers and the conditions under which they work and live. Typically, they live with other men in barracks, separated from their wives, children, and extended family. This lack of social support can create situations of extreme anxiety and depression for farmworkers, compounded by their isolation and poverty, and exacerbated by the challenges of identity that spans two cultures.

Fear of violence and retribution causes unique stress on migrant farmworkers that may not be experienced by other immigrants. Farmworkers' lives are often tightly controlled by contractors, the middle men between the growers and the farmworkers, who determine when they are paid, how much, when they arrive and leave the workplace, the type of housing and transportation they have access to, along with other basic services. Contractors are associated with some of the most severe abuses in agriculture, as they control every aspect of daily life of the farmworker [22]. Once farmworkers arrive in Southern Georgia, rather than starting with a clean slate and earning money to send back to their families in Mexico or support their families in the United States, many begin with the burden of repayment of the debt of passage to the United States.

Traditional Latino gender norms intersect with the isolation of farmwork to make for a very lonely life for many of the male farmworkers in Southern Georgia. Jackie describes explaining to the Family Farmworker Health Project students that male farmworkers might present to the students with what seems like a minor injury, and they are looking for caring and emotional support that they do not often get in their daily lives, as many are separated from their mothers, wives, and children. In addition, traditional gender norms adversely impact women, who are often expected to prepare food for men in the adjacent barracks, while also working in the fields or packing sheds for 10 to 12 hours per day. Jackie commented that if a woman ends up in a domestic violence shelter, the goal of the shelter is to assist women in becoming self-sufficient, which requires employment. If the woman is undocumented, she cannot become employed, which creates a grave problem for undocumented women who are abused. Jackie also speculated that sometimes men would prefer that their female partners remain undocumented as a way of maintaining power over the household.

While the majority of farmworkers in southern Georgia are undocumented, women are even more likely to be undocumented than men, as they may have joined their spouses in Georgia who are on H2A visas illegally [23]. Women who migrate with their partners bear unique vulnerability to mental illness and violence due to the gendered power difference within the family [24]. In addition, the fear of deportation often creates anxiety and depression. Jackie reported a very high level of domestic violence within farmworker families. If the couple has differing legal status, this may prevent women who are abused from seeking help due to many barriers, including a language barrier, lack of autonomy, and fear of deportation. While there is a way of receiving temporary documentation if a woman is being abused (a U visa), substantial paper work and legal representation are required [25].

## 4. Conclusion

As with all case studies, our ability to generalize the findings of this case study to other cases is limited. While the results of this case study identify critical themes of migrant farmworker health among a small, rural population of farmworkers in southern Georgia, the salience of invisibility, isolation, and limited access to healthcare has been noted in several previous studies. Garcia and Gondolf identified situational factors that increase problem drinking among Mexican farmworkers as social isolation and peer influence [26]. They concluded that programs and policies are necessary to support migratory families and offset social isolation as a means to address problem drinking [26]. Hovey and Magana, in several publications on mental health among Mexican migrant farmworkers, acknowledge the impact of social isolation resulting from physical isolation as negatively impacting their mental health [8, 9]. Isolation from family and friends results in feelings of anguish [27]. Hovey and Magana further expands upon the theme of isolation and mental health. He names “geographical and social isolation” as represented by stressors associated with being physically isolated. He also describes “emotional isolation” as characterized by the emotional inability to confide in others [9, page 114].

There are several important limitations to consider in this study. The possibility of selection bias exists in this case study—the group of farmworkers represented may have unique characteristics that brought them to the United States, and also in contact with Jackie. It is important that future studies be conducted that employ a variety of methodologies to strengthen the body of knowledge of migrant farmworker health, which in turn will inform interventions and policy decisions.

As this case study shows, the health of migrant farmworkers in southern Georgia is influenced by many factors. Farmwork is one of the top three most dangerous occupations in the United States [28]. Moreover, the risks of agricultural work for Latino migrant farmworkers go beyond the physical dangers of the job; both their physical and mental health are at increased risk. Multiple theories have been proposed to understand the health of migrant workers, with several focusing on the health of migrant farmworkers in particular [8]. While certain aspects of these theories capture elements of the health concerns voiced in this case study, they all fail to capture the impact of *isolation* from family and community, as well as the *invisibility* of farmworkers before institutions.

Years of participation and the narratives of the key informant in this study highlight the lived experience of migrant farmworkers in Georgia. Their reality constitutes an important challenge to the dominant theory of acculturation on several levels of analysis. In order to adopt American beliefs and behaviors, farmworkers would need access to institutions utilized by Americans, such as churches, health care facilities, the judicial system, and domestic violence shelters. Due to fears of deportation, lack of English skills, and transportation, farmworkers are unable to access these resources. The isolation experienced by farmworkers is exacerbated by their lack of access to health care. Despite significant disease burden and excess mortality rates from certain diseases and injuries, farmworkers utilize health care less frequently than others [29]. Without sufficient access to health care, farmworkers suffer from the late stages of preventable and treatable illness. In fact, even for the H-2A legally documented farmworkers, there is no screening for infectious or chronic disease.

 Acculturation theory assumes that economic integration facilitates cultural integration. While farmworkers certainly have skills that are necessary for a successful harvest—like correctly picking a tomato plant so that it continues to flower—their skills are not transferable. Paradoxically, acquiring skills limits the economic possibilities of farmworkers, as successfully integrated migrants fill niche positions within labor markets, whereas farmworkers' skills are tied to a unique market where self-sufficiency is difficult [30].

The insider perspective on migrant farmworker health illuminates critical issues that impact their health. Farmworkers are invisible before the law; they lack the protections afforded to workers in every other sector. While H-2A workers are entitled to some benefits, such as workers' compensation, at least 75% of farmworkers and up to 90% of children of farmworkers do not have health insurance [31]. Migrant farmworkers occupy a liminal space that challenges traditional definitions of group membership.

This paper has endeavored to illustrate why farmworkers remain trapped in a self-perpetuating liminal location and, thus, cannot advocate for themselves. Nurses and other health professionals are called to actively advocate for a more socially just policy towards working conditions and access to educational and healthcare institutions that could improve their physical and mental health. Much of the American discourse surrounding immigration policy addresses the strain immigrants put on our social systems, educational system, and health care system. Nurses can contribute their voices to the immigration discourse as well, so that policy makers can understand the reality of the farmworker's life. Farmworkers play an essential and irreplaceable role in American food production, and they deserve to be seen and heard.

Nurses who care for farmworker populations are uniquely positioned to provide on-the-ground witness to the lived experience of migrant workers. Such accounts make visible the scope of the human condition within state borders; the intersubjectivity that nurses can relate to wider audiences, can in this way contribute to “the engine of social change” [20]. This paper is a contribution by nurses to make visible the subjective experience of migrant farmworkers in the case of Georgia.
